# On the Frequent Occurrence of Indigo in Human Urine, and on Its Chemical and Physiological Relations

**Published:** 1853-11

**Authors:** Arthur Hill Hassall

**Affiliations:** Physician to the Royal Free Hospital, &c.


"*On the frequent occurrence of Indigo in Human Urine, and on its Chemical and Physiological Relations.*" By ARTHUR HILL HASSALL, M. D. Physician to the Royal Free Hospital, &c.

[The following is an abstract of a very interesting paper, by Dr. A. H. Hassall, read at one of the late meetings of the Royal Society, London. Its publication may, perhaps, induce others to repeat Dr. H.'s investigations, with the object of defining more especially than he has done, the diseases in which the blue indigo occurs, so that the discovery may be applied to a practical purpose in diagnosis.] S. L.

The author was led to the investigations laid before the Society in the above communication, by the following circumstances:—

Some three or four years since, when examining urinary deposits under the microscope, he frequently observed in the field of vision, particles of a deep blue color; so often did this occur that he could not even then help suspecting that their presence was not accidental; however, no analysis of the blue coloring matter was made at the time, and the circumstance was in a fair way of being forgotten, until the recollection of it was revived by another occurrence.

In June 1852, a sample of urine, freely exposed to the air in an open vessel, was observed after four or five days, exposure, gradually to change color; the pellicle or scum which had formed upon the surface of the urine became first slate-color, and at length deep blue, with here and there a rust-red tint. The urine also underwent at the same time some remarkable changes; it became thick and turbid, deep brown, greenish, bluish-green, and finally of a faded yellowish-green color; a considerable sediment was found at the bottom of the glass; this was deep brown, intermixed with a little blue coloring matter, and it had a medicinal smell resembling somewhat that of *Valerian.* In this state, without undergoing any further material changes, the urine remained for many days.

Examined with the microscope, the scum or pellicle on the surface was found to consist of vibriones, innumerable animalcules, and crystals of triple phosphate, with a great many fragments and granules of a deep and bright blue color.

A second sample of the same urine was therefore procured, every precaution being taking to avoid fallacy. Gradually the same changes ensued as in the first sample, and this likewise became blue. Having thus ascertained that the changes observed were due to something contained in the urine itself, the author next proceeded to set aside in open vessels a series of urines all from the same patient, noting the alterations which occurred from day to day; these samples underwent nearly similar changes, but the quantity of blue coloring matter and brown extractive gradually diminished, until at length they were present in such small amount as to be visible only under the microscope, and at last they entirely disappeared.

The result obtained from an examination of the *urine*, the *blue coloring matter* and the *brown extractive*, are then given by the author; they are as follow:—

*The urine.*—The urine of the second sample at the time of analysis, when shaken, had a dark greenish-brown color; but on standing at rest for some time, the coloring matter fell to the bottom, forming bluish-green flocculi, while the supernatant liquid was of a deep wine-red color. The bottle was set aside corked, for ten days, at the end of which time the bluish-green precipitate had entirely disappeared; but on removing the cork and allowing free access of air for some time, the colored deposit was again produced. This was washed with water, drenched with hydrochloric acid, and finally dried; by this means was obtained a rich blue powder possessing all the chemical characters and properties of indigo.

The urine that was filtered off from the above precipitate was allowed to evaporate spontaneously, by which means it yielded an additional quantity, which adhered in the form of very small flakes to the sides of the dish. It also gave a rather large proportion of a deliquescent brown coloring matter, which was treated, first with alcohol, and then with water.

The alcohol acquired a deep brownish-red color, and the water a dark brownish-red green. Both of these solutions were evaporated at a temperature of 160° Fahr. The alcoholic solution furnished a rich brown extractive which was soluble in water, but not in dilute acids, and nitric acid did not produce that play of colors which is characteristic of bile-pigment; nor did the precipitate formed with basic acetate of lead furnish a purple liquid with alcohol and free acid. A strong solution of potash dissolved the extractive and yielded a deep blood-red fluid, which was rendered green and opalescent by boiling. These reactions show that the brown pigment was somewhat like hæmatine in its chemical manifestations.

While the aqueous solution of the brown matter was undergoing evaporation, it gave a further supply of indigo, which was formed most freely at the edge of the liquid. The residue was made black by concentrated sulphuric acid, and deep brown by potash.

*The blue coloring matter.*—Of two samples of this in a dry state, mixed with a large quantity of earthy phosphates, vibriones, mucus, and epithelium, one gave a dark brown solution with concentrated sulphuric acid, and the other a dirty blue. Both of these solutions were decomposed by water, furnishing in the former case a dark brown deposit, and in the latter a dirty green. In most of their other reactions, however, they presented the characters of indigo; and it is especially deserving of notice, that they were reduced by lime and sugar, giving a liquid from which hydrochloric acid threw down a greenish-blue precipitate.*

*The brown extractive.*—The brown extractive yielded nearly the same results as on its first analysis, and the aqueous solution furnished a few blue flocculi. A portion of the alcoholic extract was heated with Liq. Potassæ for the purpose of ascertaining whether it contained leucine, and the product, on being treated with hydrochloric acid, gave off a powerful odor, which was somewhat like *valerianic acid;* but the result was too doubtful to be of much value. The author had already referred to the peculiar smell of *Valerian* emitted by the extractive of more than one of

* The cause of concentrated sulphuric acid giving with one of these samples a brown solution, and with the other only a dirty blue, was, the author considers, mainly owing to the large quantity of animal matter with which the specimens were contaminated; the acid from its charring effect on this, would produce a brown, or blackish solution, thus obscuring the color of the solution of indigo.

the samples. He considers that the clearest and most positive evidence was thus obtained that the blue coloring matter in this case was indigo.

It was not very long after the occurrence of the first case of blue urine that numerous other instances fell under the author's observation. The urines of all these cases underwent very nearly the same changes as in the first; in some the quantity of blue coloring matter found was very considerable, in others less; and in the third class of cases the microscope was necessary for its discovery. In nearly all these cases the blue coloring matter was submitted to analysis and ascertained, on the clearest evidence, to be indigo.

The author in the next place considers the question of the source and origin of indigo in the urine.

It appeared that in the cases related by the author, colored indigo was not present in the urine when first voided, but that it was gradually formed some time afterwards by a process of oxidation or exposure to the air, being in most of the cases probably derived from the brown extractive, which in its chemical manifestations so closely resembles hæmatine.

The author contrasts *cyanourine* with the indigo detected in uiine. He observes that the most distinctive tests laid down for cyanourine are its solubility in boiling alcohol, and the action of sulphuric acid upon it, which gives a reddish-brown solution; and states that he had ascertained that these tests are not to be relied upon, since indigo, when contaminated (as in the urine it frequently is) with a large quantity of animal matter, vibriones, &c., gives a reddish-brown solution with concentrated sulphuric acid, from the charring of the animal matter, and in many cases forms a bright blue solution with boiling absolute alcohol; hence he could not help suspecting that cyanourine and indigo are very closely connected with each other, if they be not identical. He observes, it is at least singular that while so many cases of indigo were met with, not a single instance of cyanourine presented itself. He also contrasts indigo with apoglancine, and remarks that this is acknowledged by Heller himself to be nothing more than cyanourine mixed with urrhodine.

Take then into consideration the whole of the facts described in this communication, and the followiug conclusions are deducted:

1st. That blue indigo is frequently formed in human urine, the quantity being subject to the greatest variation; in some cases it is so considerable as to impart a deep green, or bluish-green color to the whole urine; a pellicle of nearly pure indigo also extending over the entire surface of the liquid; while in others it is so small that it can only be detected by means of the microscope.

2d. That for the formation of this indigo, it is in general necessary that the urine should be exposed to the air for some days in an open vessel, oxygen being absorbed and the blue indigo developed. Whatever, therefore, facilitates oxygenation, a free exposure to light and air, warmth and sunshine, hastens the appearance of the indigo; hence in summer the changes described take place much more quickly than in winter; on the contrary, the changes are retarded and even stayed by the exclusion of the atmosphere. Blue indigo may even be deprived of its color and reformed, alternately, according as air is excluded or admitted to urine containing it. From some of the cases recorded, it would appear, however, that blue indigo is occasionally formed in the system, and is voided as such in the urine.

3d. That there is usually found with the blue indigo, where the amount of this is considerable, a brown extractive, sometimes in large quantity, the aqueous solution of which, by exposure to air, yields a further supply of colored indigo, and which closely resembles hæmatine in its chemical manifestations and elementary composition. There is therefore great reason for believing that in the majority of the cases here recorded, the blue indigo was derived from altered hæmatine, although it is at the same time probable, that in some cases it is formed from modified urine pigment, which is itself supposed to be a modification of hæmatine. Between the greater number of the animal coloring matters there is the closest relationship in chemical composition, so that the transformation of the one into the other would appear to be both easy and natural.

4th. That the urines in which the colored indigo occurs in the largest quantity, are usually of a pale straw color, readily becoming turbid, alkaline, and of low specific gravity. Small quantities of indigo are however found in urines possessing characters the very reverse, that is, in such as are high-colored, acid, and of high specific gravity; but as a rule, in these urines the blue pigment is usually absent.

5thly. That as colored indigo does not occur in healthy urine, and since, where the amount of this is at all considerable, it is accompanied with strongly-marked symptoms of deranged health, the promotion of blue indigo in urine, must be regarded as a strictly pathological phenomenon, apparently associated rather with some general morbid condition, than essentially with disease of any one organ; although there is reason for believing that the blue deposit is met with very frequently in Bright's disease, and in affections of the organs of respiration, it should however be remarked that none of the worst cases of indigo in the urine which the author met with, were cases of Bright's disease.

The paper is illustrated by drawings, and a specimen of the indigo, as deposited from urine, was exhibited.—*Philosophical Magazine*, Sept. 1853.

